# Prospective multicenter feasibility study of omnipolar near-field peak-frequency mapping for slow-pathway localization in typical atrioventricular nodal re-entrant tachycardia

**DOI:** 10.1186/s12872-026-06779-5

**Published:** 2026-10-19

**Authors:** Marzia Giaccardi, Stella Cartei, Valentina Mangiafico, Maria Lucia Narducci, Roberto Scacciavillani, Marina Zampieri, Maria Luisa Loricchio, Carlos Alberto Centurión Aznaràn, Bruno Albano, Lina Marcantoni, Ambra Del Greco, Giulia Brizzi, Benito Baldauf, Gemma Pelargonio

**Affiliations:** 1 Cardiology Florence II, Department of Medical Specialties, Florence, Italy; 2 Pediatric Cardiology Unit, Meyer Children’s Hospital IRCCS, Florence, Italy; 3 Department of Cardiovascular and Thoracic Sciences, Catholic University of the Sacred Heart, Rome, Italy; 4 Department of Cardiology, Sandro Pertini Hospital, Rome, Italy; 5 Santa Maria Della Misericordia Hospital, Rovigo, Italy; 6 Abbott Medical, Viale Thomas Alva Edison 110, Sesto San Giovanni, 20099 MI Italy; 7 University of Applied Sciences, An der Karlstadt 8, 27568 Bremerhaven, Germany; 8 Medical Faculty, University of Kiel, Christian-Albrechts-Platz 4, 24118 Kiel, Germany

**Keywords:** Avnrt, Catheter ablation, Omnipolar technology, Peak frequency, Electroanatomic mapping

## Abstract

**Background:**

Slow-pathway (SP) ablation is the established therapy for atrioventricular nodal re-entrant tachycardia (AVNRT). While conventional anatomical and electrogram-guided approaches are effective, SP localization may be challenging in a subset of procedures. Omnipolar near-field (OTNF) peak frequency (PF) mapping has been proposed as a signal-based approach to support SP localization; however, prospective multicenter data remain limited.

**Methods:**

Fifty consecutive adults with inducible typical AVNRT underwent catheter ablation in three Italian hospitals. High-density right atrial mapping was performed during sinus rhythm using a 16-pole grid catheter (Advisor™ HD Grid) with OTNF on the EnSite X system. PF maps were generated (high-frequency threshold > 250 Hz), and RF applications were delivered to the region of highest PF. Voltage maps were also created to delineate low-voltage bridge (LVB) regions (typically 0.04–2 mV) for retrospective overlap/localization analyses; LVB was not used to select ablation targets.

**Results:**

Acute procedural success was achieved in all patients, with no procedural complications. The effective ablation site (junctional beats with subsequent SP modification and/or non-inducibility) corresponded to the highest PF region in 45/50 patients (90%). In a retrospective overlap/localization analysis, the effective site fell within the LVB region in 36/50 patients (72%); concordance between PF-defined and LVB-defined regions at the effective site was observed in 35/50 patients (70%). Mean PF in the SP target region was 424 ± 86 Hz versus 272 ± 57 Hz in surrounding tissue (*P* < 0.05 on paired testing). Exploratory ROC analysis yielded an AUC of 0.937 with an optimal cutoff of 357 Hz (sensitivity 0.87; specificity 0.94); these estimates should be interpreted cautiously because PF guided RF target selection (incorporation/target-selection bias).

**Conclusion:**

In this prospective multicenter cohort, OTNF-derived PF mapping was feasible and reproducibly identified a discrete region used to guide SP ablation in typical AVNRT. ROC-derived thresholds and performance estimates are exploratory and require independent validation in cohorts where ablation targets are not selected using PF criteria.

**Supplementary Information:**

The online version contains supplementary material available at https://doi.org/10.1186/s12872-026-06779-5.

## What’s New?

- In this prospective multicenter cohort of 50 patients with typical AVNRT, omnipolar near-field peak-frequency (PF) mapping was feasible, with acute success in all patients, no procedural complications and no recurrences at one year.
- The effective slow-pathway ablation site corresponded to the highest-PF region in 90% of patients, and mean PF was higher in the target region than in surrounding tissue (424 ± 86 Hz vs 272 ± 57 Hz).
- The effective site fell within the low-voltage bridge in 72% (retrospective overlap analysis only). The exploratory PF cutoff of 357 Hz needs independent validation, because PF guided target selection.
- Potential utility in non-inducible AVNRT remains hypothesis-generating and warrants dedicated investigation.

## Introduction

Atrioventricular nodal re-entrant tachycardia (AVNRT) is the most common form of supraventricular tachyarrhythmia. The established therapeutic approach involves ablation of the slow pathway (SP), located within the triangle of Koch (TK) [1]. Traditionally, successful SP ablation has been guided by anatomical landmarks and electrophysiological criteria under fluoroscopic guidance. Advancements in electroanatomic mapping (EAM) systems enable more precise localization of relevant substrates and may reduce fluoroscopy exposure. Radiofrequency ablation (RFA) remains the standard treatment, with procedural success defined by junctional beats and/or non-inducibility of AVNRT, with or without atrioventricular nodal echo beats [2, 3].

Despite high success rates and a favorable safety profile [4], SP localization may be challenging in a subset of procedures. Several mapping techniques have been proposed to improve localization, including voltage mapping [5, 6], isochronal late activation mapping [7], and targeting fractionated signals [8, 9]. Voltage mapping is commonly used because the SP has been described as a “low-voltage bridge” (LVB), a region of low-amplitude myocardium connecting areas of higher voltage [5]. However, LVB identification can be difficult and may vary with voltage thresholds and mapping conditions [10].

In addition to voltage-based approaches, near-field and peak-frequency–based mapping strategies have been reported to support SP localization. In this study, we evaluated the feasibility and multicenter reproducibility of OTNF PF mapping integrated in the EnSite X system [11]. The OTNF algorithm automatically measures and annotates the highest PF detected in mapped intracardiac electrograms (EGMs), generating a frequency map that depicts the distribution of higher-frequency activity.

The objective of this prospective multicenter study was to assess the feasibility and reproducibility of PF-guided SP localization in typical AVNRT. We additionally performed a retrospective overlap/localization analysis between PF-defined regions and LVB regions, without a head-to-head comparison of ablation strategies.

## Materials and methods

Patients were eligible if aged ≥ 18 years and undergoing electrophysiological study with catheter ablation for symptomatic AVNRT confirmed during the procedure (October 2024–June 2025). Patients were prospectively enrolled. Inclusion required documented supraventricular tachycardia on ECG monitoring or clinical symptoms highly suggestive of AVNRT (recurrent palpitations, dizziness, presyncope, or syncope). Only patients with inducible AVNRT during electrophysiological testing and complete procedural and follow-up data were included. The study was conducted at three Italian hospitals: Santa Maria Annunziata Hospital (Florence), Policlinico Gemelli Hospital (Rome), and Sandro Pertini Hospital (Rome). Baseline characteristics are summarized in Table 1.

**Table 1:** Baseline population characteristics

	Overall (N=50)
Age (years)	57 ± 16
Female gender (n,%)	32 (64)
Hypertension (n,%)	12 (24)
Dyslipidaemia (n,%)	12 (24)
Diabetes (n,%)	3 (6)
Prior ablation procedure (n,%)	1 (2)
Left ventricular ejection fraction (%)	59 ± 4
Values are presented as mean ± SD or as number (%).	

Antiarrhythmic drugs were discontinued at least 3 days before the procedure. After informed consent, all patients underwent EAM using the Advisor HD Grid Sensor Enabled catheter with OTNF. Standard catheter placement included a decapolar catheter in the coronary sinus and two quadripolar catheters (right atrial appendage and His bundle region). Programmed atrial pacing was performed to assess dual AV nodal physiology and to induce sustained AVNRT.

### Mapping and ablation workflow

Voltage and frequency mapping were performed during sinus rhythm using the HD Grid catheter with OTNF. The voltage color scale was adjusted individually to highlight LVBs, typically within 0.04–2 mV. Multiparametric maps overlaying voltage and PF data were generated using the Emphasis™ module. An initial PF cutoff of 250 Hz was applied to define near-field components [12]. RF energy was delivered to the region showing the highest PF. LVB maps were generated for retrospective overlap/localization analyses and were not used to select ablation targets.

Because the total mapping point dataset (mean 9040.8 ± 6702 points per procedure) encompassed the entire right atrium, including valvular, appendage, caval, and ventricular points collected automatically during geometry reconstruction, points within the SP region of interest could not be isolated through automated export. For each patient, 10 mapping points within the SP region were therefore manually selected based on EGM morphology characteristic of AVNRT and anatomical location; this selection process was applied consistently across all 50 patients.

RFA was performed using irrigated ablation catheters (Tactiflex™ SE, Abbott) with a power setting of 30 W, temperature controlled at 55 °C, and irrigation at 2 mL/min. Ablation targets included contact force > 5 g and impedance drop ≥ 10 Ω. Procedural success was defined by elimination or modification of SP conduction and/or non-inducibility of AVNRT [13]. All procedures were performed with a zero-fluoroscopy approach, adhering to the ALARA principle [14].

### Statistical analysis

Categorical variables are expressed as number (%). Continuous variables are reported as mean ± SD or median (IQR), as appropriate. Within-patient comparisons (e.g., frequency and mapped area in the SP target region versus surrounding tissue) were analyzed using paired Student’s t-tests when the distribution of within-patient differences was approximately normal, or Wilcoxon signed-rank tests otherwise. The unit of analysis for the logistic regression and ROC analyses was the mapping point (500 observations from 50 patients; 10 electrograms sampled per patient within the SP region). These models were fitted using a standard binomial generalized linear model and did not include an adjustment for within-patient clustering; as such, they should be interpreted as exploratory, hypothesis-generating results rather than clustering-adjusted inferential estimates. ROC analyses were additionally considered exploratory because PF guided RF target selection introduces incorporation/target-selection bias. Within-patient comparisons of frequency and mapped area (paired analyses described above) were unaffected by these limitations, as each patient contributed a single paired observation. All analyses were performed using R version 4.4.2 (R Foundation for Statistical Computing, Vienna, Austria). A two-sided P value < 0.05 was considered statistically significant.

## Results

Fifty consecutive patients (mean age 57 years; 64% women; Table 1) underwent EAM-guided SP ablation for typical AVNRT with right inferior extension (RIE). AVNRT was confirmed by electrophysiological study in basal conditions and during isoproterenol infusion. Dual AV nodal physiology was defined by an AH “jump” (≥ 50 ms increase in A2H2 following a 10 ms decrement in S1S2 coupling interval).

Mean total procedure time was 86.8 ± 19.5 min (including a 20-minute observation period). Mean mapping time was 9.3 ± 5.4 min. The mean number of total mapping points collected was 9040.8 ± 6702 (Table 2).

**Table 2:** Procedure and mapping characteristics

Procedure features	
Procedure time (min)	86.8 ± 19.5
Mapping time (min)	9.3 ± 5.4
Electroanatomic map	
Total mapping points	9040.8 ± 6702
Used mapping points	2294.5 ± 3322.8
Effective ablation site corresponded to highest PF region (junctional beats elicited)	45/50 (90%)
Effective ablation site falling within LVB region (overlap/localization analysis)	36/50 (72%)
Concordance between PF-defined and LVB-defined regions at the effective ablation site	35/50 (70%)
Ablation	
Number of Automark	6 ± 4
Total ablation time (sec)	120 ± 32
Total ablation time on HF area of the LVB (sec)	81 ± 14
RF power (W)	30 ± 4

Values are presented as mean ± SD or as number (%).

PF mapping was used to guide RF delivery in all patients (Supp. Figures 1, 2). In a retrospective overlap/localization analysis, the effective ablation site corresponded to the highest PF region in 45/50 patients (90%) and fell within the LVB region in 36/50 patients (72%). Concordance between PF-defined and LVB-defined regions at the effective site was observed in 35/50 patients (70%) (Table 2; Figs. 2 and 3).

**Fig. 1:**
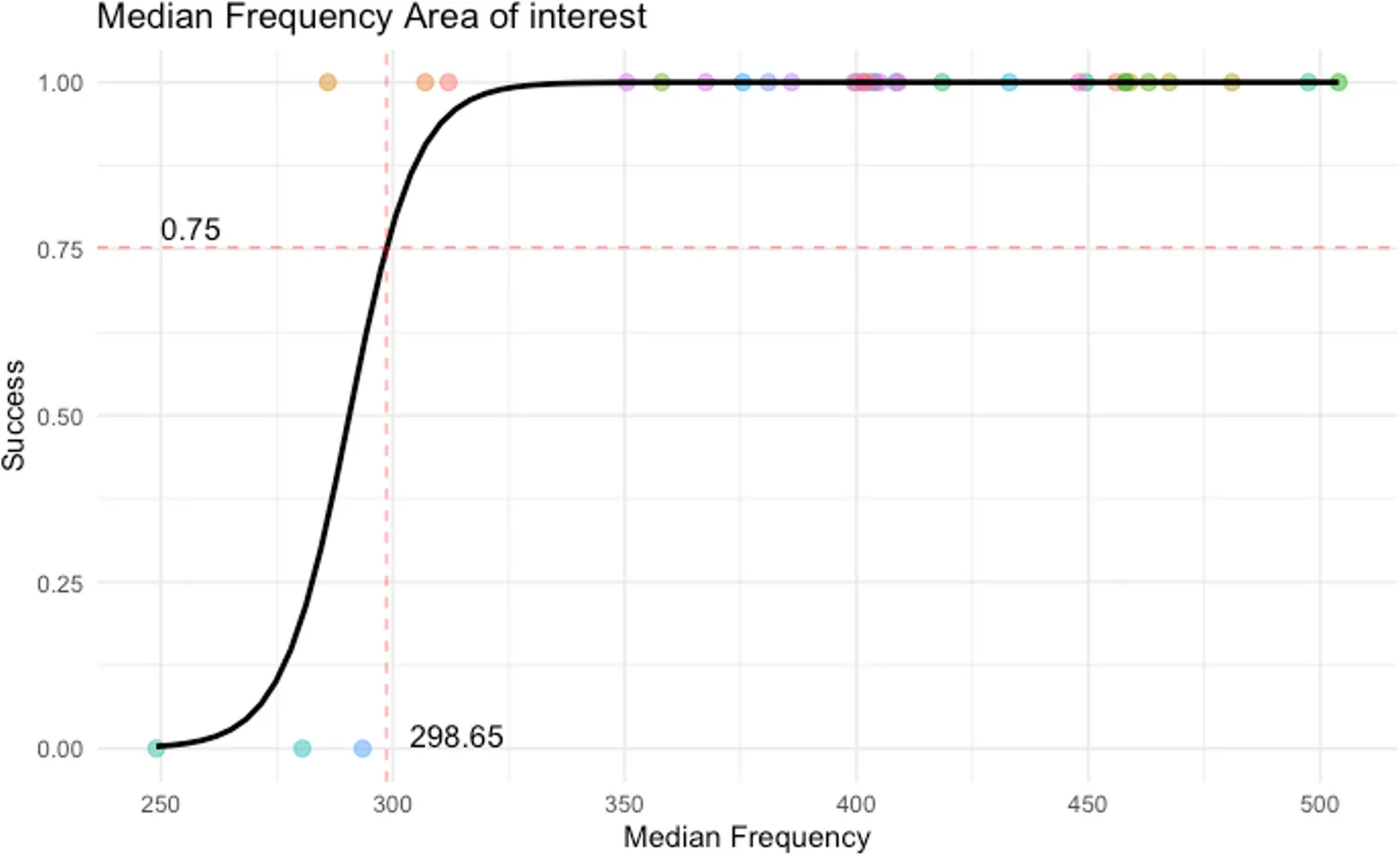
Within-patient comparison of Peak Frequency Area and Low Voltage Bridge Area. Box plots show the distribution of the mapped areas. Differences between measurements were assessed using the paired Wilcoxon signed-rank test. The PF-defined high-frequency area was significantly smaller than the mapped LVB area (P < 0.05)

**Fig. 2:**
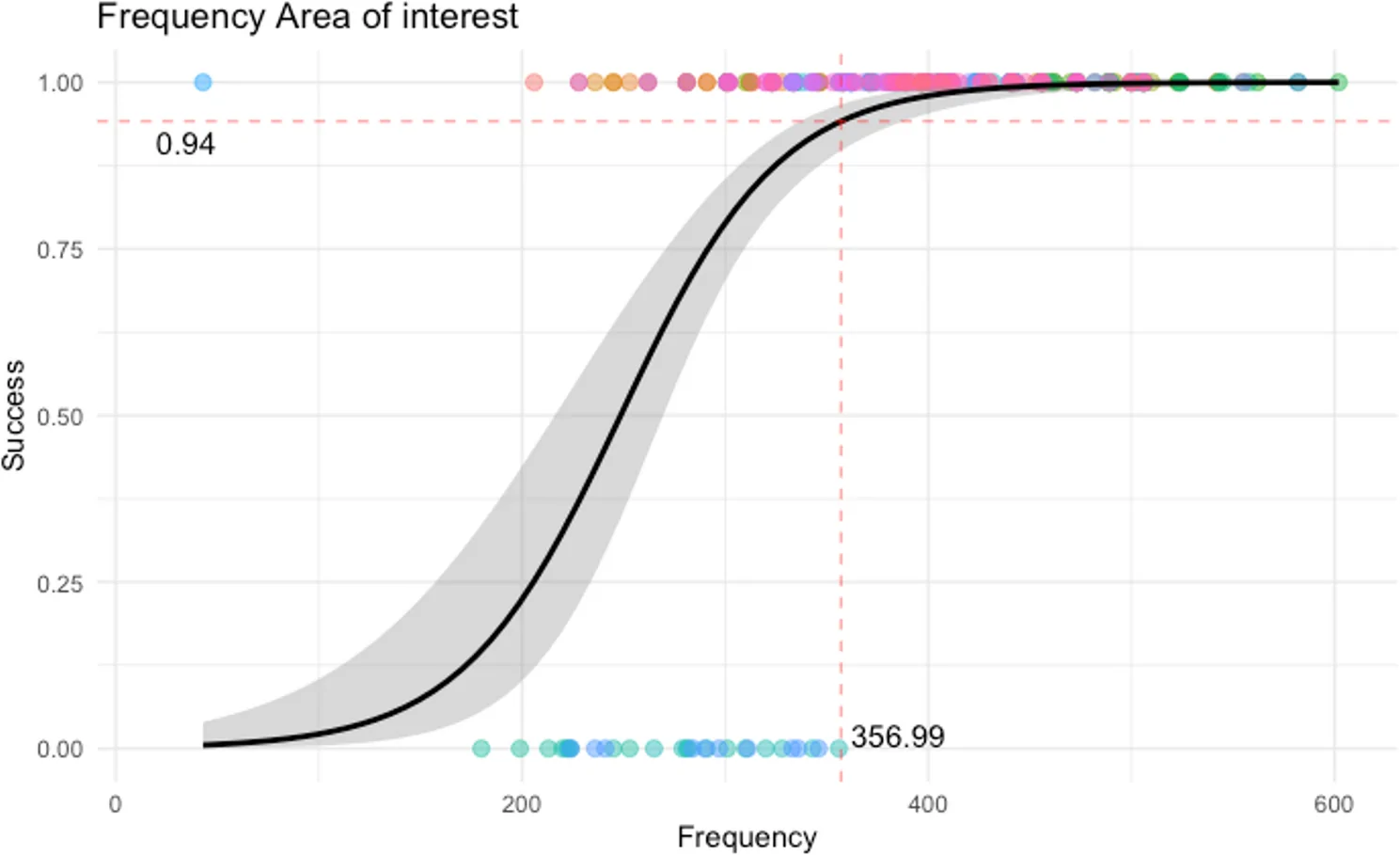
High resolution mapping of the triangle of Koch in LAO and LL views during sinus rhythm. Omnipolar voltage map (0.04–0.5 mV) with PF overlay (cutoff >250 Hz). Overlaying PF data refines the ablation target to a smaller region

**Fig. 3:**
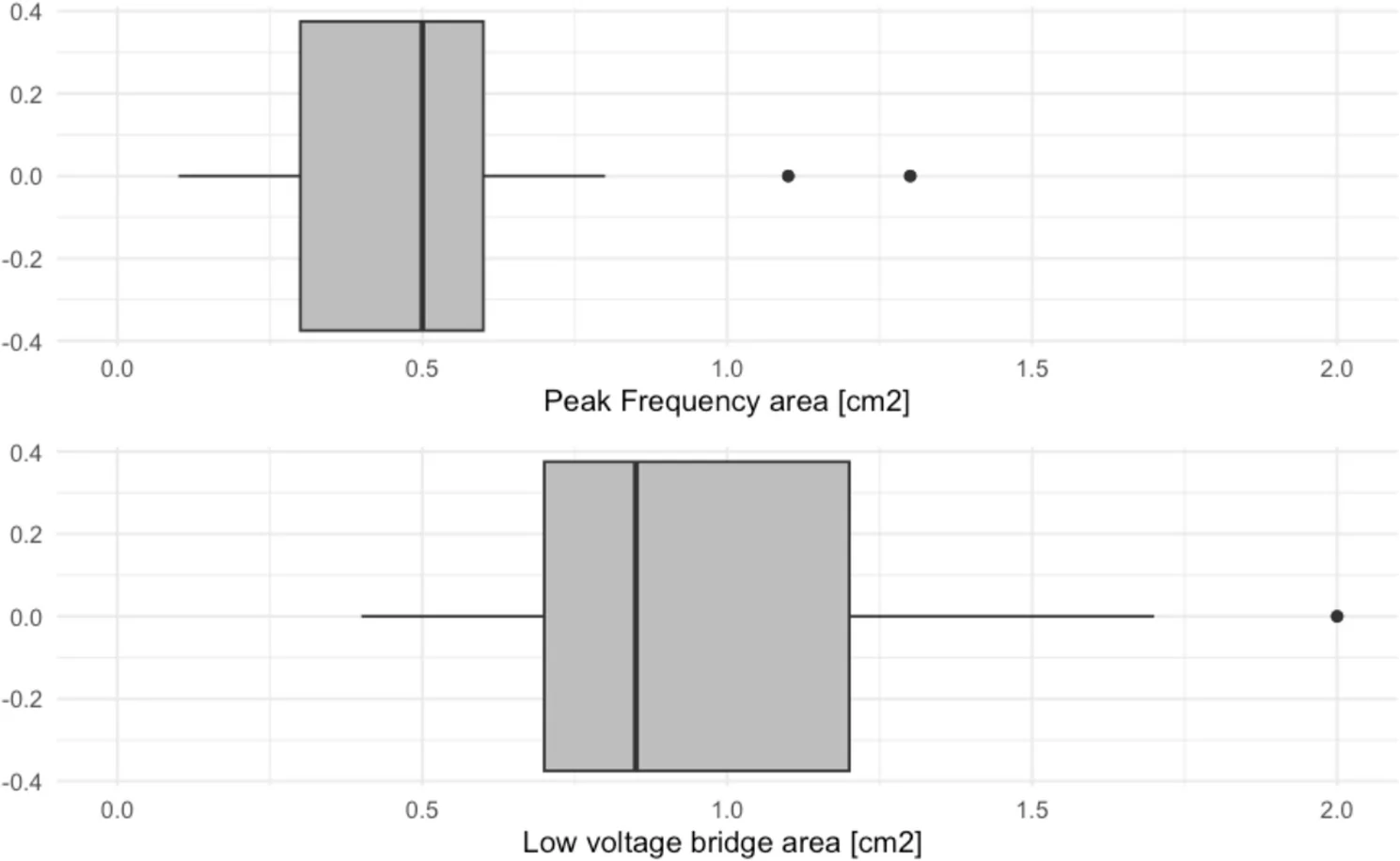
High resolution mapping of the triangle of Koch in LL views during sinus rhythm. Left: omnipolar voltage map depicting the LVB area. Right: PF map highlighting the highest frequency region. The figure illustrates that the LVB region does not always overlap with the effective PF targeted site where junctional beats were elicited (overlap/localization analysis)

The mapped LVB area measured 1.0 ± 0.4 cm², whereas the PF-defined high-frequency area measured 0.5 ± 0.3 cm². Within-patient comparison using the paired Wilcoxon signed-rank test demonstrated a significant difference between the two areas (*P* < 0.05) (Table 3; Fig. 1).

**Table 3:** Within patient comparison of frequency and mapped area between the SP target region and surrounding tissue

LVB			
	SP area	Around SP area	p value
Frequency (Hz)	424 ± 86	272 ± 57	<0.05
Area (cm^2^)	HF on LVB 0.5 ± 0.3	LVB 1.0 ± 0.4	<0.05

Values are presented as mean ± SD

**Fig. 4:**
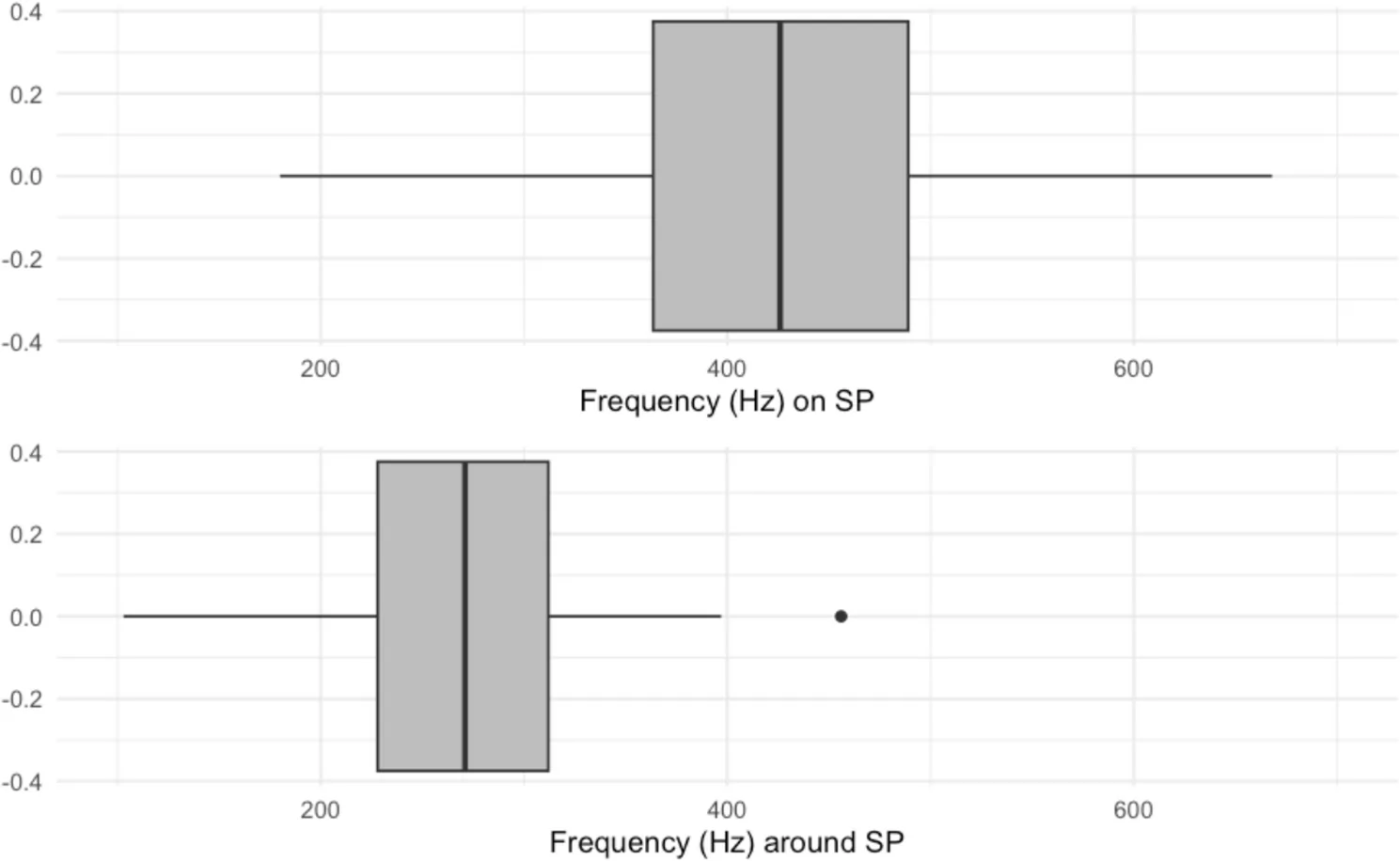
Within-patient comparison of peak frequency values measured within the SP region and in the adjacent area. Box plots display the distribution of peak frequency values. Peak frequency was significantly higher within the SP region than in the surrounding area (P < 0.05). Differences between measurements were assessed using a paired Student's t-test

Mean PF within the SP target region was 424 ± 86 Hz compared with 272 ± 57 Hz in the surrounding tissue. Within-patient comparison using a paired Student’s t-test demonstrated significantly higher PF values within the SP region (*P* < 0.05) (Table 3; Fig. 4).

Ablation required a mean of 3.0 ± 1.19 RF applications, with a total RF time of 120 ± 32 s, of which 81 ± 14 s were applied to the high-frequency region.

**Fig. 5:**
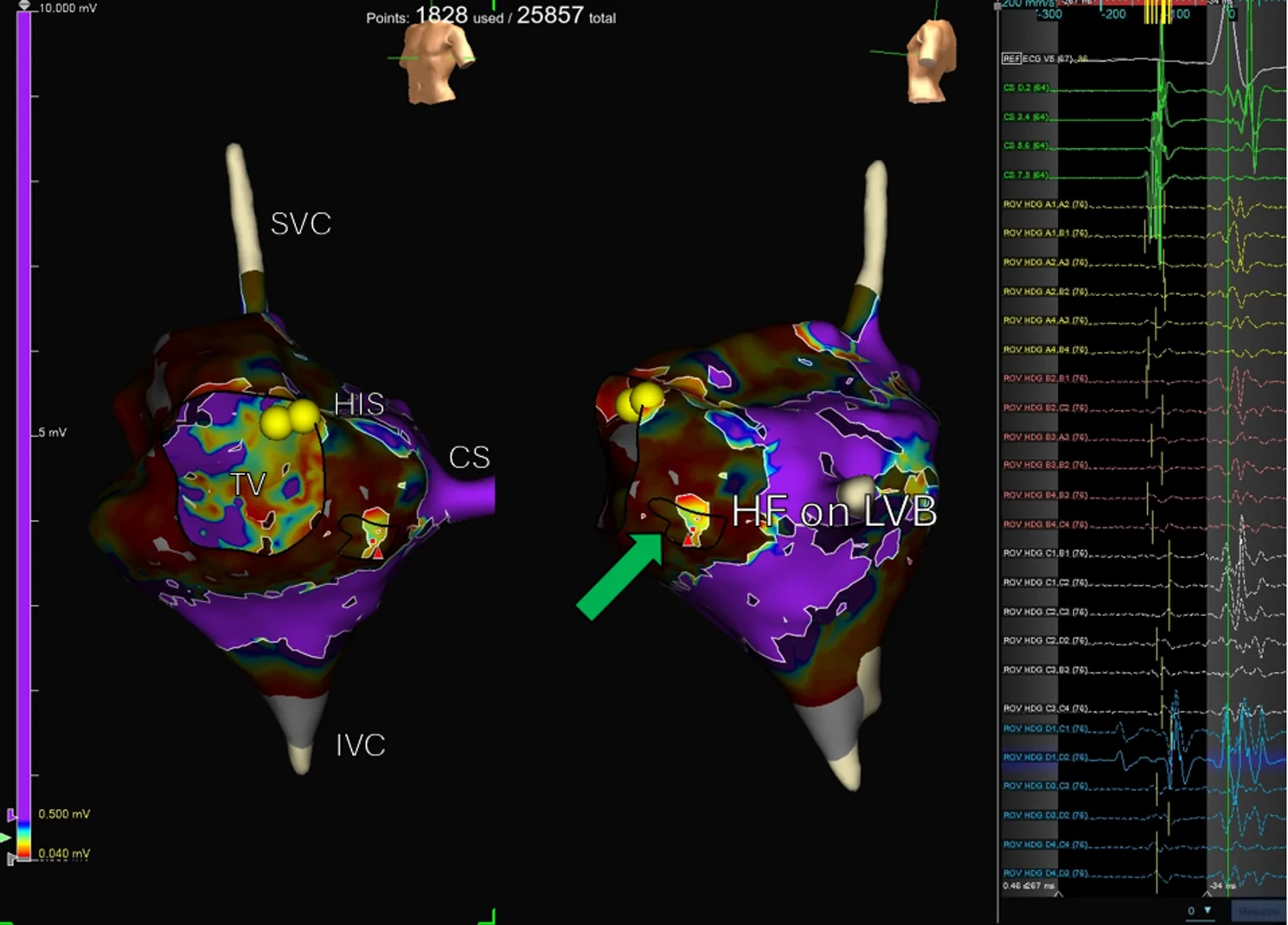
Exploratory logistic regression model using maximum PF to describe the relationship between PF metrics and identification of the effective SP target

**Fig. 6:**
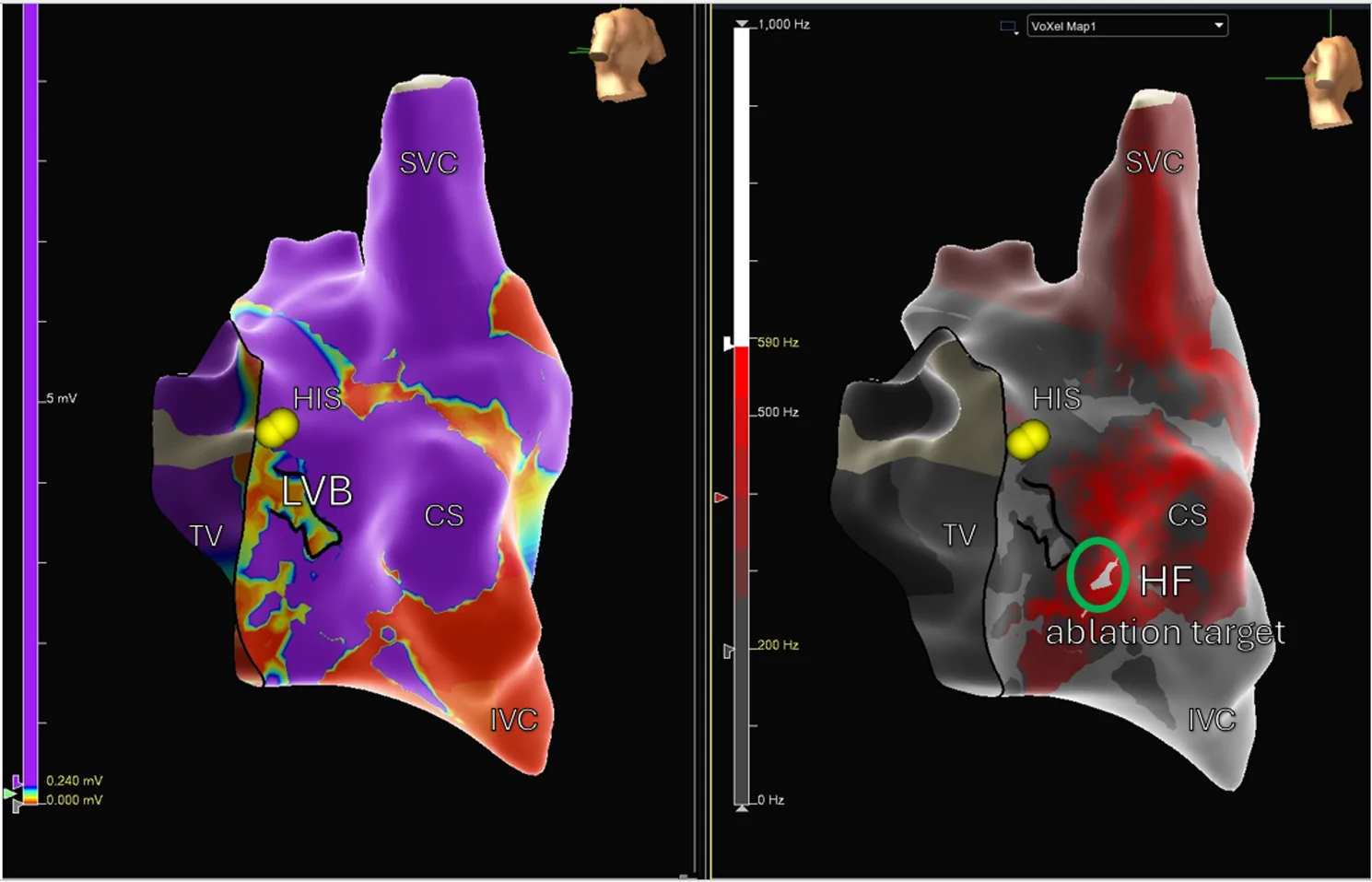
Exploratory logistic regression model using median PF to describe the relationship between PF metrics and identification of the effective SP target

**Fig. 7:**
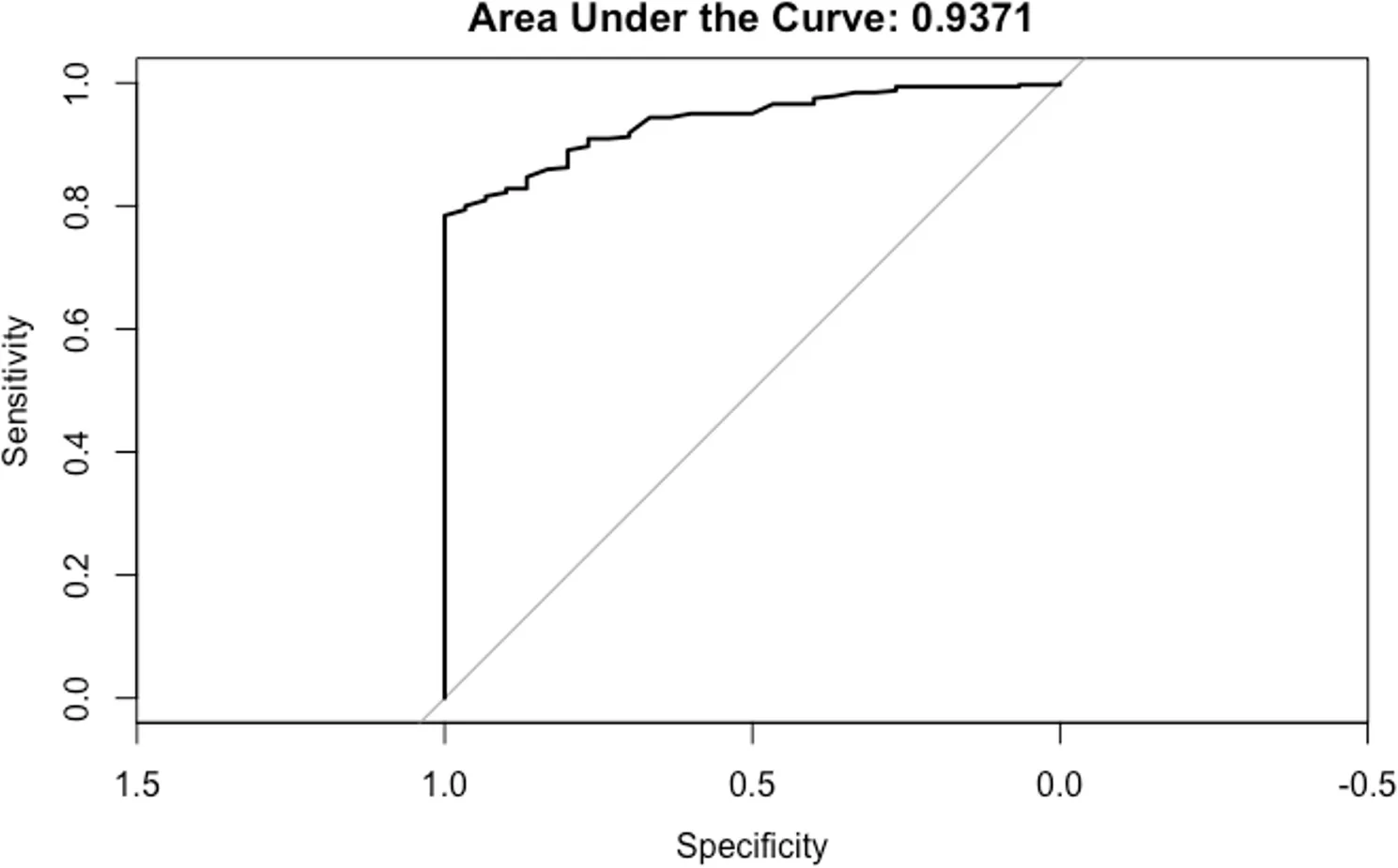
Exploratory ROC curve evaluating PF metrics for identification of the effective SP target (AUC = 0.9371). ROC results should be interpreted cautiously due to incorporation/target selection bias because PF guided RF target selection

Exploratory logistic regression and ROC analyses (500 mapping points from 50 patients) were performed to describe the relationship between PF metrics and identification of the effective SP target (Figs. 5 and 6). The optimal PF cutoff in this dataset was 357 Hz, and the ROC AUC was 0.9371 (Fig. 7); these estimates should be interpreted cautiously due to incorporation/target-selection bias.

Count of PF areas, low voltage bridge areas, frequency on and around SP can be found on supplementary Tables 1–4.

No procedural complications were reported. Post-ablation testing confirmed SP modification/elimination in all cases, with no residual jumps or echo beats observed. After one year of follow-up, clinical success was 100%, with no AVNRT recurrences reported.

## Discussion

Haïssaguerre potentials (high-low frequency signals) are typically recorded just inferior to the AV node, above the tricuspid annulus (TA) [15]. They likely reflect asynchronous activation of deep atrial cells (high-frequency component) and superficial nodal cells near the TA (low-frequency component), corresponding to the presumed RIE. Inoue et al. proposed that the SP includes the RIE and leftward inferior extensions and clarified aspects of the re-entry circuit [16]. The RIE, located along the inferior aspect of Koch’s triangle between the coronary sinus ostium and the TA, serves as a substrate for SP conduction [17] and provides the antegrade limb in typical AVNRT [18].

In this prospective multicenter cohort, OTNF-derived PF mapping during sinus rhythm was feasible and provided a discrete region used to guide SP ablation. The PF-defined region corresponded to the effective ablation site (junctional beats with subsequent SP modification and/or non-inducibility) in 90% of patients. In a retrospective overlap/localization analysis, the effective site fell within the LVB region in 72% of patients; this does not represent the performance of an LVB-guided ablation strategy because LVB was not used to select RF targets.

We observed higher PF in the SP target region compared with surrounding tissue. Although we report an AUC of 0.9371 and an optimal cutoff of 357 Hz, these findings should be considered exploratory because PF guided RF target selection; therefore, incorporation/target-selection bias may inflate performance estimates. Independent validation is required in cohorts where ablation targets are not PF-selected.

Previous studies reported favorable outcomes of SP ablation using voltage gradient mapping in adult and pediatric patients [5, 19–21]. However, delineating the LVB can be challenging and may be influenced by operator-dependent threshold selection and anatomical variability [10]. PF mapping may provide an automated, signal-based visualization that may support localization of a discrete ablation target region [22]. Our findings are consistent with those reported by Takahashi et al. [23], who observed comparable PF values at successful SP ablation sites.

Given the low complication rates and high procedural success of conventional SP ablation techniques [24], the incremental clinical impact of PF mapping will need to be evaluated in comparative studies. The potential utility of PF mapping in non-inducible AVNRT remains hypothesis-generating and warrants dedicated investigation.

## Limitations

This study has several limitations. Inter-operator variability and differences between centers may have influenced mapping acquisition and annotation. Our findings are limited to adult patients with inducible typical AVNRT and may not generalize to pediatric patients or atypical AVNRT substrates. The use of isoproterenol could have affected recorded signals.

Importantly, ROC and cutoff analyses are subject to incorporation/target-selection bias because PF guided RF delivery; therefore, AUC/cutoff/sensitivity/specificity estimates are exploratory and may overestimate independent diagnostic performance. Furthermore, the 10 mapping points selected per patient for these analyses were chosen based on EGM morphology characteristic of AVNRT and anatomical location rather than through blinded or randomized sampling; this non-blinded selection represents an additional potential source of bias, distinct from but compounding the target-selection bias described above. In addition, the logistic regression and ROC analyses were performed at the mapping-point level (500 observations from 50 patients) without adjustment for within-patient clustering, which may have led to underestimation of standard errors for these exploratory analyses.

## Conclusion

In this prospective multicenter study of typical AVNRT, OTNF-derived PF mapping during sinus rhythm was feasible and reproducibly identified a discrete region used to guide SP ablation. ROC-derived thresholds and related performance estimates should be interpreted as exploratory because PF guided RF target selection, and independent validation is required in cohorts where ablation targets are not PF-selected.

## Supplementary information

Below is the link to the electronic supplementary material.

Supplementary Material 1

## Data Availability

All data generated or analyzed during this study are included in this published article.

## Abbreviations

AAD: Antiarrhythmic drug
AVNRT: Atrioventricular nodal re entrant tachycardia
EAM: Electroanatomic mapping
HF: High frequency
LVB: Low voltage bridge
OT: Omnipolar technology
OTNF: Omnipolar technology near field
PF: Peak frequency
SP: Slow pathway
SVT: Supraventricular tachycardia
RFA: Radiofrequency ablation
RF: Radiofrequency
TK: Triangle of Koch
