# Supplementary material for: Prospective multicenter feasibility study of omnipolar near-field peak-frequency mapping for slow-pathway localization in typical atrioventricular nodal re-entrant tachycardia
Source: BMC Cardiovasc Disord. 2026 Oct 19;26:973. doi: 10.1186/s12872-026-06779-5 (PMC13695299; doi:10.1186/s12872-026-06779-5)
Supplement: Supplementary file 1 [file 12872_2026_6779_MOESM1_ESM.docx]

**Supplements:**

**Supplementary Table 1.** Peak Frequency area counts

**Supplementary Table 2.** Low voltage bridge area counts

**Supplementary Table 3.** Frequency values on the slow pathway

**Supplementary Table 4.** Frequency values around the slow pathway

**Supplementary Figure 1.** High-resolution mapping of the Koch triangle in Left Lateral views during sinus rhythm. The image displays on the left the omnipolar voltage map ranging from 0.00 to 1.257 mV showing the LVB area. The right panel shows the Peak Frequency map, with the highest frequency highlighted in white, using a cutoff above 410 Hz within the slow pathway region. This figure illustrates LVB mapping with PF overlays for identification of junctional beats.

**Supplementary Figure 2.** High-resolution mapping of the Koch triangle in Left Lateral view during ablation with Tactiflex catheter. The image displays the Peak Frequency (mixed-effects models) map, with the highest frequency highlighted in white, using a cutoff > 590 Hz within the slow pathway region. This figure illustrates how the Peak Frequency map guides the ablation strategy by indicating sites where junctional beats are elicited during the procedure.
